# Aluminium-Based Plasmonic Sensors in Ultraviolet

**DOI:** 10.3390/s21124096

**Published:** 2021-06-14

**Authors:** Karol Karpiński, Sylwia Zielińska-Raczyńska, David Ziemkiewicz

**Affiliations:** Institute of Mathematics and Physics, UTP University of Science and Technology, Aleje Prof. S. Kaliskiego 7, 85-796 Bydgoszcz, Poland; karol.karpinski@utp.edu.pl (K.K.); sziel@utp.edu.pl (S.Z.-R.)

**Keywords:** surface plasmons, ultraviolet, nanostructure

## Abstract

We theoretically investigate the surface plasmon polaritons (SPPs) generated on an Al film covered by an Al_2_O_3_ layer in the context of their application as refractive index sensors. The calculated reflection spectra indicate SPP resonance excited by ultraviolet light, which was affected by the thickness of both the metal and the oxide layers on the surface. With optimized geometry, the system can work as a tunable sensor with a wide UV wavelength range λ∼ 150–300 nm. We report a quality factor of up to 10 and a figure of merit on the order of 9, and these are comparable to the performance of more complicated UV plasmonic nanostructures and allow for the detection of a 1% change of the refraction index. The sensor can operate on the basis of either the incidence angle or wavelength changes. The effect of oxide surface roughness is also investigated with an emphasis on amplitude-based refraction index sensing.

## 1. Introduction

Surface plasmon polaritons (SPPs) are localized electromagnetic wave modes that propagate at the interface between a metal and dielectric material. The excitation of SPPs is possible only under specific conditions regarding the exciting light wavelength, the incidence angle, and the refraction index of the material surrounding the metal layer. This latter property makes them particularly useful in sensing applications.

The electric field of SPPs is highly localized, which enables the miniaturization of plasmonic devices [1]. Typically, the main design challenge of such devices is in overcoming the absorption [2], which results in a widespread use of noble metals, especially in the spectral range of visible light. However, the usual materials cannot be used beyond visible wavelengths [3]. On the other hand, aluminium is a promising candidate for UV plasmonics due to its particularly large bulk plasma frequency [4]. With the appropriate geometry, low absorption and considerable propagation distances can be achieved [5].

There are many promising application fields of UV plasmonics, such as biomedical imaging [6,7], thickness measurement [8], photoacoustics, electrochemistry [9], and label-free DNA and single molecule sensing [9,10]. Due to the fact that many media exhibit a significant absorption in the UV region, an SPP sensor in this spectral range may be characterized by particularly good material selectivity and sensitivity [11]. Currently, with the rapid development of technology and the need for specialized plasmonic sensors in the ultraviolet wavelength region, aluminium has become a favoured metal for designing such devices with its combination of low cost and ease of fabrication [6,12,13].

Here, we investigate the performance of Al-based plasmonic sensors in the near ultraviolet spectral region. One of the characteristic features of Al is the native oxide layer that appears when the Al film is exposed to air. This, as a consequence, influences the UV-SPPs sensing performance. The integration of a passive oxidation layer into the device design not only allows for its operation in normal atmospheric conditions but may also be used for the detection of oxidizing agents, such as ozone [14]. Due to the fact that the thicknesses of both the Al film and Al_2_O_3_ layers affect the sensor performance, it is crucial to optimize the geometry of the sensor.

In contrast to the nanoparticle- [6] and nanostructure- [8] based approaches, we started from the basic case of a continuous metal layer that was easy to fabricate. The oxidation layer inherent to the Al surface was taken into account as an integral part of the structure. In the experimental study by Tanabe and Tanaka [11], the reflectance of an Al surface was found to be consistent with a theoretical model that assumed a fixed 4-nm oxide layer. Here, we present a general theoretical and numerical approach using the thickness of the Al_2_O_3_ as one of the optimization parameters of the plasmonic sensor and indicating how the oxide layer thickness can be optimized for specified operating conditions. Moreover, the possibility of enhancing the sensitivity by a modification of the geometry of the oxide surface is explored.

The paper is organized as follows. In the first section, the basic theory of SPP propagation is presented, and the reflection coefficient of a four-layer system is derived. Then, the details of finite-difference time-domain simulation are described, with a particular focus on modelling the optical properties of the dispersive media in the system. In the next section, theoretical and numerical results are presented. Following that, the results are discussed and compared with the experimental data. Finally, in the last section, our conclusions are presented.

## 2. Theory

The SPPs are localized electromagnetic excitations that occur on an interface between two media that exhibit opposite signs of dielectric permittivity for some frequency ω. Usually the material with negative permittivity $\epsilon_{1}\left(\omega \right)$ is a metal, and the one that is characterized by $\epsilon_{2}\left(\omega \right)>0$ is dielectric. Such a system is described with the help of the Maxwell’s equations with boundary conditions at the metal–dielectric interface. In solving this set, one obtains the wave vector $\vec{k}$ of the SPP. Particularly, a plasmonic mode propagating along the interface is characterized by a component parallel to the interface [1]

(1) $$\kappa \left(\omega \right)=\kappa^{\prime }+i\kappa^{\prime \prime }=\frac{\omega }{c}\sqrt{\frac{\epsilon_{1}\epsilon_{2}}{\epsilon_{1}+\epsilon_{2}}},$$

where $\kappa^{\prime }$ and $\kappa^{\prime \prime }$ are, respectively, the real and imaginary parts of the wave vector component, and *c* is the vacuum light velocity. For real values of $\epsilon_{1}$ and $\epsilon_{2}$, one obtains a real wave vector when $\epsilon_{1}\epsilon_{2}<0$ and $\epsilon_{1}+\epsilon_{2}<0$. In such a case, propagating SPPs can be excited. However, an effective excitation is possible only when the parallel wave vector component of the incident light matches κ; this puts limits on the incidence angle α. The permittivity ϵ is directly related to the refraction index $n=\sqrt{\epsilon }$.

**Figure 1:**
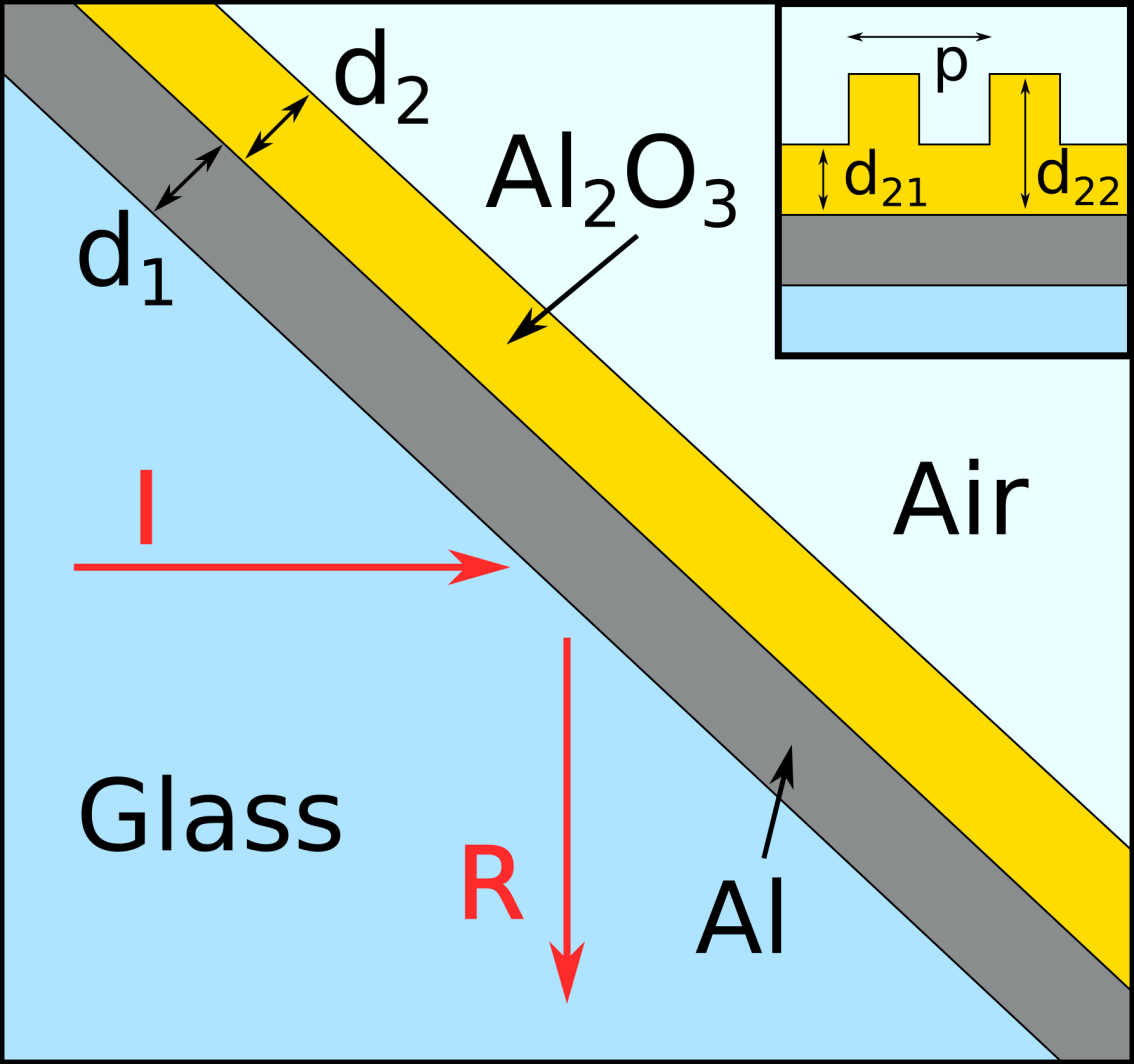
Schematic representation of the investigated system. Inset: the overall structure of the grating on the top of the oxide layer.

In this paper, we examined a plasmonic sensor in a form of a four-layer structure with the general geometry as shown in Figure 1. The setup follows the Kretschmann configuration [15], where the incident light *I* propagates in a glass prism (characterized by the permittivity $\epsilon_{1}=2.25$). The light on an Al layer of the thickness $d_{1}$ is at the angle of incidence α∼45 degrees. The third layer is aluminium oxide (Al_2_O_3_) with the thickness $d_{2}$.

Finally, the last medium is either air or some material characterized by the permittivity $\epsilon_{4}$, which is measured by the sensor. The wavelength values referred to throughout the manuscript are the free space wavelengths of the source λ=2πc/ω, where ω is the source frequency. The wavelength of the surface plasmon is shorter by a factor $n_{SPP}=\frac{\kappa^{\prime }c}{\omega }$, where $n_{SPP}$ is the effective refraction index of the plasmon, which can be derived from its dispersion relation k(ω).

For SPPs in the ultraviolet range (λ∼200 nm), one has $n_{SPP}∼2.5$. We consider a wide range of Al and Al_2_O_3_ layer thicknesses; the starting point was the system presented in [11], where a 19-nm Al layer with a natural 4-nm oxide layer was used. The performance of thicker aluminium layers up to 44 nm is investigated. For the Al_2_O_3_, we consider values up to $d_{2}=16$ nm, which can be produced with electron beam evaporation [16].

One can calculate the reflectivity *R* of *N* parallel layers system using Parratt’s recursive method, which treats the system as a stratified medium on top of an infinitely thick substrate [17]. We assume that the substrate and the surroundings are semi-finite and that the inner materials have finite thickness $d_{i}$. Each layer is described by the dielectric function $\varepsilon_{i}$, where *i* = 1, …, N + 1. The dielectric functions are described by the Drude–Lorentz model

(2) $$\varepsilon \left(\omega \right)=\varepsilon_{\infty }+\sum_{j=1}^{J}\frac{\omega_{pj}^{2}}{\omega_{0j}^{2}+i\omega \gamma_{j}-\omega^{2}}$$

which describes the optical properties of the medium in terms of harmonic oscillators (*j* = 1, 2, …, *J*) with plasma frequencies $\omega_{pj}$, resonance frequencies $\omega_{0j}$, and damping constants $\gamma_{j}$. These parameters were obtained by fitting the permittivity given by Equation (2) with two oscillators to the experimental data [5]. In particular, we use the data for Al [4] and Al_2_O_3_ [18], while the permittivity of glass is assumed to be a constant $\varepsilon_{1}=2.25$, and the permittivity of air is $\epsilon_{4}=1$. In the simulations where the refraction index sensing is tested, air is replaced with another medium with $\epsilon_{4}>1$. The fitted model values are shown in Table 1.

The value of the wave vector of a plane wave with angular frequency ω, propagating in the medium *i* (*i* = 1, …, 4) is given by

(3) $$k_{i}=\frac{n_{i}\omega }{c}$$

where $n_{i}$ is the refraction index of the medium *i*. For a given incidence angle α, the components of wave vector parallel to surface $k_{x}$ and perpendicular $k_{z}$ are given by

(4) $$\begin{array}{c} k_{x}=\frac{\omega }{c}\sqrt{\varepsilon_{1}}\sin\alpha , \end{array}$$

(5) $$\begin{array}{c} k_{zi}=\sqrt{\varepsilon_{i}\frac{\omega^{2}}{c^{2}}-k_{x}^{2}}. \end{array}$$

Using these values, one can derive the Fresnel coefficient,

(6) $$F_{i}=\frac{\frac{k_{z_{i}}}{\varepsilon_{i}}-\frac{k_{z_{i+1}}}{\varepsilon_{i+1}}}{\frac{k_{z_{i}}}{\varepsilon_{i}}+\frac{k_{z_{i+1}}}{\varepsilon_{i+1}}},$$

for each interface between the layers *i* and i+1. Additionally, each of the layers of thickness $\eta_{i}$ introduces a phase shift $\phi_{i}=e^{2ik_{z_{i}}\eta_{i}}$. In the system described in this paper, there are only two layers of the finite thicknesses, marked $d_{1}$ and $d_{2}$: the metal characterized with permittivity $\epsilon_{2}$ and thickness $\eta_{2}=d_{1}$ and the oxide layer characterized by $\epsilon_{3}$, $\eta_{3}=d_{2}$. The values of $\eta_{1}$ and $\eta_{4}$ are taken as 0 due to the fact that the phase shifts $\phi_{1}$ and $\phi_{4}$ in these media are irrelevant to the solution. Parratt’s recursive relation yields

(7) $$\begin{array}{c} R_{i}=\phi_{i}\frac{F_{i}+R_{i+1}}{1+F_{i}R_{i+1}}. \end{array}$$

The recurrence relation may be solved for $R_{0}$, which then provides the total reflectivity of the system $R=|R_{0}{|}^{2}$. Since the last layer is infinite, there is no reflection at its end, and hence $R_{4}=0$. In the presented four-layer case, one can obtain an analytical solution

(8) $$R={\left|\frac{F_{1}+\phi_{2}\frac{F_{2}+\phi_{3}\frac{F_{3}}{1+F_{3}}}{1+F_{2}\phi_{3}\frac{F_{3}}{1+F_{3}}}}{1+F_{1}\phi_{2}\frac{F_{2}+\phi_{3}\frac{F_{3}}{1+F_{3}}}{1+F_{2}\phi_{3}\frac{F_{3}}{1+F_{3}}}}\right|}^{2}.$$

## 3. Numerical Simulations

The Finite-Difference-Time-Domain method is a valuable tool in the analysis of plasmonic systems. The method consists of solving Maxwell’s equations in a finite region that is divided into a spatial grid of discrete values. An in-house MATLAB code from our previous work [19] was adapted to the simulation of a four-layer structure. Expressions for time evolution of the electric and the magnetic field were derived and integrated with finite time step.

The spatial simulation domain used in this work was two-dimensional. The whole domain was divided into a rectangular grid with a single cell size δx. The magnetic field $\vec{H}=\left[0,0,H_{z}\right]$ was perpendicular to the plane, and the electric field $\vec{E}=\left[E_{x},E_{y},0\right]$ had two components in the plane of incidence located at every cell of the grid. Calculations of reflection spectra were performed in a square computational domain with the size of 300 × 300 cells of equal size ∆x = 4 nm, which was the minimum structure size that could be simulated.

The domain size of 1200 × 1200 nm was sufficient for the simulation of propagating waves up to λ∼400 nm before diffraction-related issues became severe. The computational domain was terminated with a 30-cell thick absorbing layer characterized by the reflection coefficient of $R∼10^{-4}$. The Auxiliary Differential Equations (ADE) method [20] based on a Drude model was used. Medium polarization $\vec{P}=\left[P_{x},P_{y},0\right]$ was computed by solving a second-order partial differential equation in the form

(9) $$\ddot{P}+\gamma_{j}\dot{P}+\omega_{0j}^{2}P=\frac{\omega_{pj}}{\varepsilon_{\infty }}E,$$

for every oscillator *j* with the fitted medium parameters: plasma frequency $\omega_{p}$, damping constant γ, and high-frequency permittivity limit $\varepsilon_{\infty }$. The full set of equations solved in the FDTD approach is as follows

(10) $$\begin{array}{c} -\mu_{0}\frac{\partial H_{z}\left(x,y,t\right)}{\partial t}=\frac{\partial E_{y}\left(x,y,t\right)}{\partial x}-\frac{\partial E_{x}\left(x,y,t\right)}{\partial y},\\ \frac{\partial H_{z}\left(x,y,t\right)}{\partial x}=-\varepsilon_{0}\frac{\partial E_{y}\left(x,y,t\right)}{\partial t}-\frac{\partial P_{y}\left(x,y,t\right)}{\partial t},\\ \frac{\partial H_{z}\left(x,y,t\right)}{\partial y}=\varepsilon_{0}\frac{\partial E_{x}\left(x,y,t\right)}{\partial t}+\frac{\partial P_{x}\left(x,y,t\right)}{\partial t}, \end{array}$$

where $P_{x}$ and $P_{y}$ are components of the polarization vector, $j_{x}$ and $j_{y}$ are the current densities in the directions *x* and *y*, and $\varepsilon_{0}$ and $\mu_{0}$ are the vacuum permittivity and permeability, respectively. Unit normalisation is used so that $\mu_{0}=\epsilon_{0}=c=1$. The above equations are rearranged to obtain time derivatives of the $E_{x}$, $E_{y}$, and $H_{z}$ fields, which are then used to calculate the field evolution with a constant time step ∆t. The used value ∆t=0.5 is the upper limit of stability of the used ADE scheme and is slightly below the upper limit of 2-dimensional FDTD ($∆t=1/\sqrt{2}$). To obtain the reflectivity at a single frequency, the simulation runs for 2500 time steps with a monochromatic source of radiation. The mean ratio of incidence to reflected power was calculated for the last 1000 steps, where steady-state conditions were established.

## 4. Results

**Figure 2:**
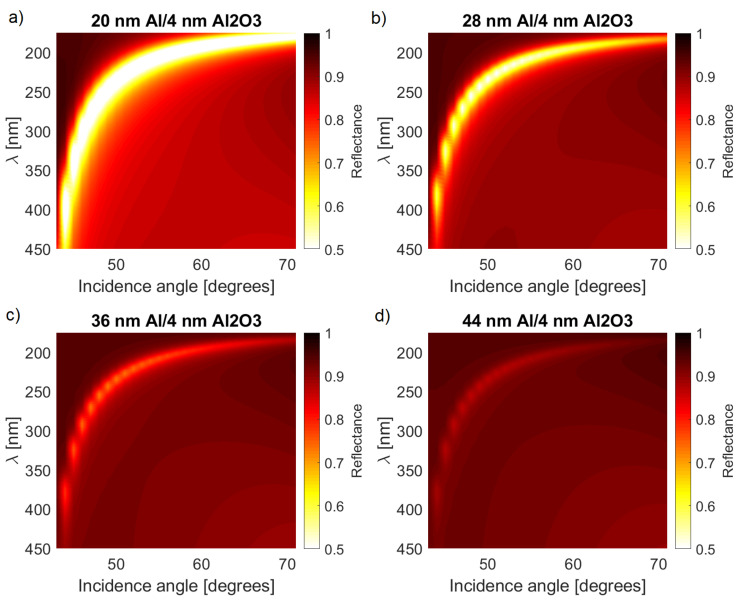
Comparison of the reflection spectra calculated for a range of wavelengths and incident angles and four different thicknesses of the metal layer: (**a**) 20 nm, (**b**) 28 nm, (**c**) 36 nm, and (**d**) 44 nm.

The first step in a design of a plasmonic sensor is the determination of the optimal conditions in which SPP resonance occurs. The main parameter that has to be determined during the fabrication process is the thickness of the Al layer. Figure 2 shows the results for different values of $d_{1}$. In general, a thicker layer results in a shallower reflection dip with a slightly smaller spectral width.

**Figure 3:**
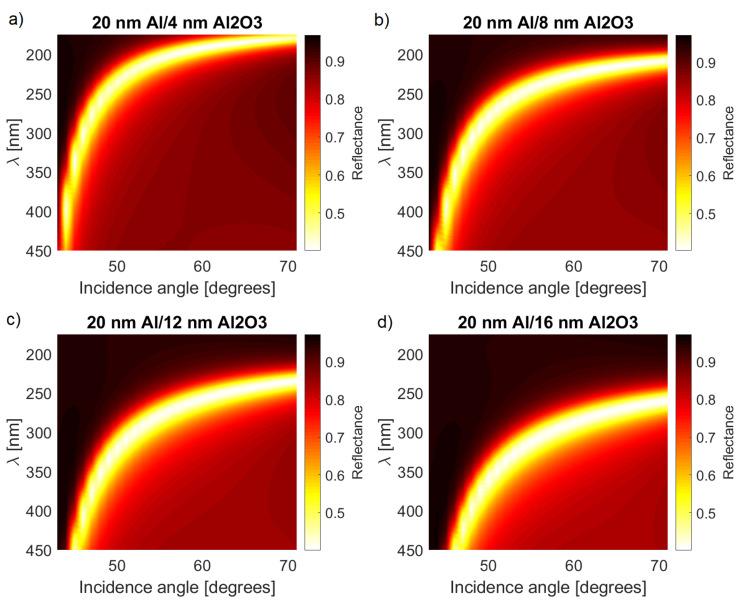
Comparison of the reflection spectra calculated for a range of wavelengths and incident angles and four different thicknesses of the oxidation layer: (**a**) 4 nm, (**b**) 8 nm, (**c**) 12 nm, and (**d**) 16 nm.

The oxidation layer that always appears on the surface of Al has a pronounced impact on the reflectivity spectrum of the metallic layer. This provides an additional degree of freedom in the sensor design due to which one can influence the width of the layer during the fabrication process. The calculation results based on Equation (8), for selected values of $d_{2}$ are shown in Figure 3. The general structure of reflectivity R(α,λ) on Figure 3a is in excellent agreement with the results presented in [11] for the comparable thicknesses $d_{1}$ and $d_{2}$. The plasmonic resonance occurred in the visible part of the spectrum at small incidence angles of α∼40 degrees, quickly moving into the UV range for α→70 degrees.

**Figure 4:**
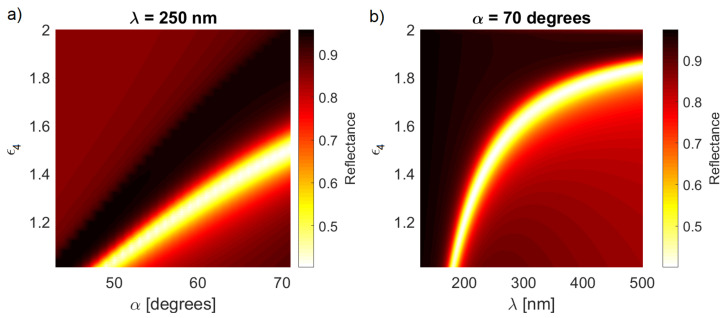
The reflection spectra as a function of the (**a**) incidence angle and $\epsilon_{4}$ and (**b**) wavelength and $\epsilon_{4}$.

The fact that the reflectivity spectrum moves significantly with the change of $d_{2}$ indicates that the system is sensitive to changes of permittivity in the near vicinity of the Al layer. This is confirmed by the results shown in Figure 4, where the spectra were calculated for $d_{1}=20$ nm and $d_{2}=4$ nm. An increase of $\epsilon_{4}$ causes a significant shift of the SPP resonance toward larger incidence angles (Figure 4a). The shift is roughly three-times greater than in the experimental results in [11] in which the refraction index (and thus permittivity) was changed only within 2 nm from the metal layer. Such a shift is also comparable to those from more complicated nanostructure-based sensors [8].

In a realistic system, one can expect some variation in the thickness of the oxidation layer. Therefore, it is useful to go beyond the simple theoretical model described by Equation (8) and perform an FDTD simulation that is based directly on Maxwell’s equations. In the first test, the multilayer system with $d_{1}=20$ nm and $d_{2}=4$ nm was illuminated by a point source of UV radiation (such as an LED diode), so that a wide range of incidence angles appear.

**Figure 5:**
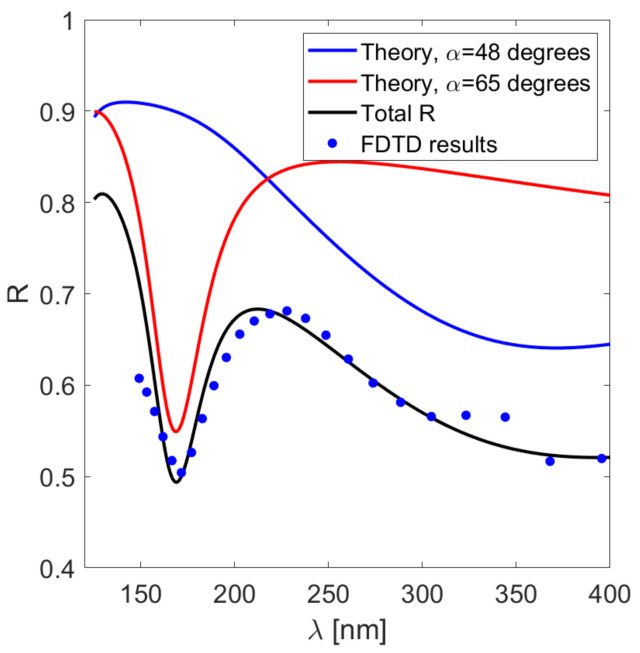
The reflectivity calculated in the FDTD simulation compared with theoretical predictions for the system from Figure 3a.

In such a case, the resulting reflection spectrum is determined by the overall structure of the reflectivity function R(α,λ) as shown in Figure 3a. As mentioned before, there are two characteristic areas in which the spectrum is independent of α and λ, located in the visible and UV parts of the spectrum. This can be seen in Figure 5 where the FDTD simulation results are presented. There are two pronounced peaks corresponding to the visible light SPPs excited at α∼48 degrees and UV SPPs at α∼65 degrees. The product of these two reflectivities closely matches the FDTD results. Thus, one can obtain significant, easily measurable reflectivity dips even when the incident angle is not precisely specified.

**Figure 6:**
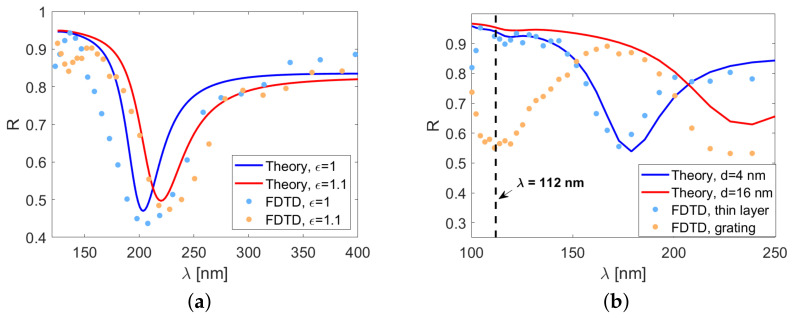
The reflectivity calculated in the FDTD simulation for (**a**) two values of $\epsilon_{4}$ and (**b**) two geometries of the oxide layer.

Next, the sensitivity of the plasmonic sensor to the changes of permittivity $\epsilon_{4}$ was tested. The simulation results are shown in Figure 6a. The incidence angle was set to α=60 degrees. Again, there was a fairly good match between the theoretical predictions and FDTD results. A 10% change of $\epsilon_{4}$ (5% change of $n_{4}$) resulted in a 20 nm shift of the reflection minimum, which is comparable to the results presented in [8].

**Figure 7:**
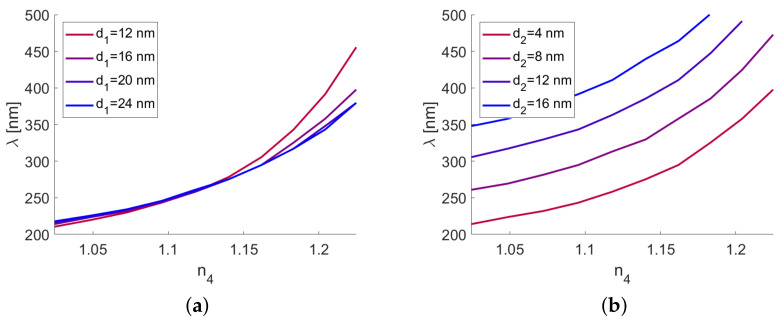
The location of the reflectivity dip as a function of $n_{4}$, calculated for (**a**) various values of $d_{1}$ and (**b**) various values of $d_{2}$.

To better assess the performance of the sensor, one can compute the shift of the reflection minimum with the change of $n_{4}$. The results are shown in Figure 7.

**Figure 8:**
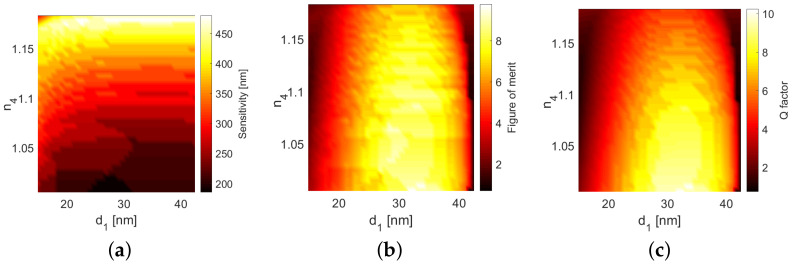
The (**a**) sensitivity *S*, (**b**) figure of merit *F*, and (**c**) Q-factor *Q* of the system as a function of $d_{1}$ and $n_{4}$.

One can see that the changes of the position of the minimum are very similar regardless of $d_{1}$, with the exception of a very small metal thickness and large $n_{4}$ (Figure 7a). The change of the oxide layer thickness $d_{2}$ resulted in a considerable shift of the minimum but had only a minimal influence on how the system responded to the changes of $n_{4}$. The rate of change of the wavelength of the reflectance dip $\lambda_{d}$ in response to the changes of $n_{4}$ was the sensitivity $S=\partial \lambda_{d}/\partial n$ [21]. To accurately resolve the location of the dip, the full-width half-maximum (FWHM) of the minimum should be small. Thus, one can introduce another important performance parameter—the figure of merit F=S/FWHM. Finally, one can calculate the Q factor $Q=\lambda_{d}/FWHM$. These parameters, calculated for an incidence angle of 60 degrees, are shown in Figure 8.

Overall, the peak sensitivity of S∼480 nm was achieved in the limit of large $n_{4}$. However, at this point, the minimum of the reflection was wide, resulting in small values of *F* and *Q*. The most well-defined minimum with the highest Q∼10, F∼9 occurred at $n_{4}∼1$, $d_{1}∼35$ nm.

Finally, one can modify the geometry of the oxide layer to improve the sensing performance. In particular, let us consider the model of a porous Al_2_O_3_ layer, which could resemble the realistic experimental situation. In particular, the form of a grating with period p=45 nm and two thickness values of $d_{21}=4$ nm and $d_{22}=16$ nm was used (Figure 1 inset). Such a structure supports the creation of plasmons with a wavelength that is a multiple of the period [19]. Here, the value of *p* was set so that SPPs could be excited with the source characterized by $\lambda =45/n_{SPP}\approx 112$ nm.

Localized SPP resonances on nanostructures [6,7] have the form of standing wave modes that may provide a superior spectral resolution or refraction index sensitivity as compared to a smooth surface. It should be stressed that, in opposition to the studies of Al nanoparticles or rough surfaces [22], here, only the oxide layer on the surface is modified, which potentially simplifies the fabrication.

The results are presented in Figure 6b. One can see that introduction of the grating gave rise to an additional reflection minimum at the wavelength equal to the grating period. Interestingly, in the grating system, the reflectivity contained the minimum corresponding to $d_{22}=16$ nm but not $d_{21}=4$ nm. The grating-related minimum was relatively narrow, comparable to that obtained for $d_{2}=4$ nm despite the fact that the effective thickness of the layer with the grating was considerably larger.

**Figure 9:**
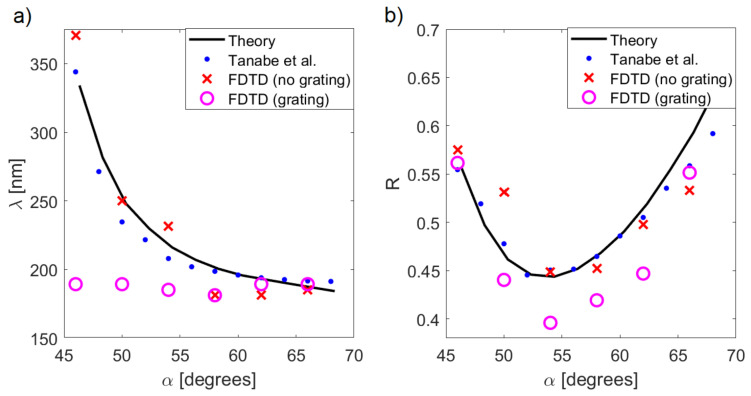
Comparison between the theoretical results, FDTD calculations, and experimental data from [11]; (**a**) the location of the reflectivity dip and (**b**) minimum value of reflectivity as a function of the incidence angle.

To further study the effect of the grating, the location of the reflectivity minimum and the smallest value of R were obtained for a range of angles α. The results are shown in Figure 9. There was excellent agreement between the theoretical predictions given by Equation (8) and the experimental data [11]; the minimum shifts were from λ≈340 nm at α=46 degrees to λ≈190 nm at α=68 degrees (Figure 9a). The largest drop of reflectivity occurred at an intermediate angle of 54 degrees, where R∼45% (Figure 9b).

The FDTD results followed the same trends, with up to a ∼10% error caused by a finite size of the numerical representation of the system; this introduced diffraction-related effects that resulted in variance of the incidence angle. When a grating with a period p=90 nm was added to the oxide layer, the excitation of plasmons by λ=190 nm source was enhanced, and thus the wavelength of the reflection minimum became tied to this geometric feature of the system and did not change with the incidence angle.

The fixed wavelength meant that the effective excitation of SPPs was more dependent on the proper incidence angle. In Figure 9b, the reflection minimum of the grating system was deeper at α∼60 degrees, which is the optimal conditions for exciting plasmons by λ=190 nm light (Figure 9a). Outside this region, the reflectivity was roughly the same as in the no grating system. This result demonstrates that the sensitivity of a plasmonic sensor based on measurement of reflection coefficient at some specified wavelength can be enhanced by an appropriately fabricated oxidation layer on top of the metal.

## 5. Discussion

The results shown in Figure 2 indicate that the optimal choice for the metal thickness $d_{1}$ depends on the desired trade-off between the magnitude of changes of *R* and the angular/spectral resolution. The angle and wavelength corresponding to the reflection minimum did not change with an increase of $d_{1}$. Additionally, with a thicker layer, the overall reflectance away from the SPP resonance approached unity.

The next important parameter is the thickness of the oxide layer. In general, a thin oxidation layer is advantageous for operation in the far-UV part of the spectrum (Figure 3). In the region of SPP resonance, one can observe an approximately 40% reduction of reflectivity, which is consistent with the experimental data [11]. By increasing the thickness of the oxide layer, one can observe a gradual shift of the reflection dip toward longer wavelengths.

The angular and spectral widths of the minimum remain unchanged. Two regions of particular interest are the areas where the spectrum is insensitive to changes of one of the parameters (α or λ). In the far UV range, for λ∼200 nm, the SPP resonance occurs in a wide range of angles α between 60–70 degrees. Conversely, for the visible part of the spectrum, the angle range is much narrower and centred around α∼45 degrees; however, the wavelength can vary in the range of 300–500 nm.

The crucial characteristic of the plasmonic sensor is its response to the changes of the refraction index $n_{4}$, which is shown in Figure 4. Both the central wavelength and the incidence angle of the reflection minimum exhibit considerable shifts for changing $n_{4}$, suggesting that both parameters are useful for the measurement of a refraction index. In particular, a small change of refraction index (∼1.5%), which corresponds to a ∼5% change of $\epsilon_{4}$, resulted in an approximately 20 nm shift of the reflection minimum when operating in the far UV regime (lower part of the Figure 4b) and up to 200 nm in the visible range. However, in the latter case, the minimum is spectrally very wide and thus it cannot be precisely located. On the other hand, the dependence of the incidence angle on $\epsilon_{4}$ is much closer to linear; the same 1.5% change of the refraction index resulted in a change of roughly 5 degrees for the optimum incidence angle.

Overall, the shift toward larger angles for a given wavelength can be intuitively interpreted; the increased permittivity $\epsilon_{4}$ serves as an effective extension of the oxide layer with $\epsilon_{3}∼3$. Similarly, in Figure 4b, there is a significant dependence of the resonant wavelength on $\epsilon_{4}$. In this particular example, the sensor was the most sensitive for $\epsilon_{4}∼1.8$; the value can be fine tuned by changing the angle or $d_{2}$, for example to adapt the design to aqueous solutions.

The FDTD results in Figure 5 and Figure 6 validate the analytical formula in Equation (8) and confirmed the theoretical predictions regarding the sensor performance. The numerically obtained minima shown in Figure 6a are only slightly wider than theoretical predictions, and the difference between $\epsilon_{4}=1$ and $\epsilon_{4}=1.1$ ($n_{4}=1$ and $n_{4}\approx 1.05$, respectively) can be easily resolved. This indicates that, with the proper choice of $d_{1}$, $d_{2}$, and λ, the variance of the incidence angle is not very detrimental to the sensor operation.

Figure 7 expands upon the results shown in Figure 4b; the roughly quadratic shift of the reflection minimum with the changes of $n_{4}$ is preserved in a wide range of thicknesses $d_{1}$ and $d_{2}$. However, while the position of the minimum remains the same for various values of $d_{1}$ (Figure 2 and Figure 7a), its size and line width change significantly, which affects performance estimates, such as the Q factor and figure of merit. These parameters, presented in Figure 8, are similar to the results presented in [21].

In particular, one can obtain either a comparable sensitivity S∼200, the figure of merit F∼9, and factor Q∼9 as in [21], or superior sensitivity S∼480 at the expense of the *F* and *Q* factor. These values can be further fine-tuned by changing $d_{2}$ and the angle of incidence. In general, *Q* and *F* are the largest for some optimal thickness of the metal layer $d_{1}$. This is consistent with the earlier observation that a thicker layer results in a smaller but narrower reflection minimum.

Finally, the simulation results obtained for the system with a grating structure of the oxide layer serve as a general indication of the behaviour of such a system, which is also closely related to realistic, rough surfaces. When the wavelength of the reflection minimum becomes mostly independent of $n_{4}$ and instead becomes fixed for the geometric features of the system, the reflectance changes are amplified. This suggests that such a geometry may be beneficial for the sensors that measure the power of the reflected light to detect changes of the refraction index [23,24].

## 6. Conclusions

We investigated the performance of an Al-based plasmonic sensor consisting of a prism, a metal layer, and an oxide layer. The effects of the variable thickness of Al and Al_2_O_3_ were studied analytically and numerically. The calculation results showed good agreement with the available experimental data and indicated how the system geometry could be optimized for a given wavelength and incidence angle of light. The theoretical performance metrics, such as the Q factor of the sensor, were calculated and compared to the available data for similar systems.

We demonstrated that the refraction index detection sensitivity could be enhanced by altering the structure of the oxide layer, which is a new approach that may prove advantageous compared to the usual metallic nanostructures in terms of the ease of fabrication. The numerical simulation confirmed the theoretical findings and indicated that FDTD can be readily used to study the reflectance of a four-layer system with complex, porous geometry.

## Figures and Tables

**Table 1:** Two-oscillator model fitting parameters (in THz).

Material	$\omega_{p1}/2\pi$	$\omega_{01}/2\pi$	$\gamma_{1}/2\pi$	$\omega_{p2}/2\pi$	$\omega_{02}/2\pi$	$\gamma_{2}/2\pi$	$\varepsilon_{\infty }$
Al	3468	0	157	0	0	0	1
Al_2_O_3_	1775	2453	263	1013	2752	335	2

## Data Availability

Not applicable.
